# Crucial Abiotic Stress Regulatory Network of NF-Y Transcription Factor in Plants

**DOI:** 10.3390/ijms24054426

**Published:** 2023-02-23

**Authors:** Han Zhang, Shujing Liu, Tianmeng Ren, Mengxue Niu, Xiao Liu, Chao Liu, Houling Wang, Weilun Yin, Xinli Xia

**Affiliations:** 1 National Engineering Research Center of Tree Breeding and Ecological Remediation, College of Biological Sciences and Technology, Beijing Forestry University, Beijing 100083, China; 2 State Key Laboratory of Tree Genetics and Breeding, College of Biological Sciences and Technology, Beijing Forestry University, Beijing 100083, China

**Keywords:** Nuclear Factor Y, abiotic stress, transcriptional regulation, functional mechanism

## Abstract

Nuclear Factor-Y (NF-Y), composed of three subunits NF-YA, NF-YB and NF-YC, exists in most of the eukaryotes and is relatively conservative in evolution. As compared to animals and fungi, the number of NF-Y subunits has significantly expanded in higher plants. The NF-Y complex regulates the expression of target genes by directly binding the promoter *CCAAT* box or by physical interaction and mediating the binding of a transcriptional activator or inhibitor. NF-Y plays an important role at various stages of plant growth and development, especially in response to stress, which attracted many researchers to explore. Herein, we have reviewed the structural characteristics and mechanism of function of NF-Y subunits, summarized the latest research on NF-Y involved in the response to abiotic stresses, including drought, salt, nutrient and temperature, and elaborated the critical role of NF-Y in these different abiotic stresses. Based on the summary above, we have prospected the potential research on NF-Y in response to plant abiotic stresses and discussed the difficulties that may be faced in order to provide a reference for the in-depth analysis of the function of NF-Y transcription factors and an in-depth study of plant responses to abiotic stress.

## 1. Introduction

Nuclear Factor-Y (NF-Y), known as the heme activator factor (*HAP*) or *CCAAT*-box binding factor (CBF), is a transcription factor (TF) ubiquitous in eukaryotes and can specifically bind to *CCAAT* cis-acting elements [1]. These proteins were named NF-Y following the suggestion of a reported study [2]. NF-Y is generally considered to be a heterotrimeric complex composed of NF-YA, NF-YB and NF-YC [3]. The intact NF-Y factor, assembled by NF-YA/B/C, mainly functions in the nucleus by directly binding to the *CCAAT* cis-acting elements in the promoter region of the target genes or by interacting with other factors to activate or inhibit the expression of the target genes [4]. Studies have shown that the animal NF-Y complex is essential in many life activities, such as cell proliferation and apoptosis, the occurrence of tumors and cancers, the stress response, growth and development [5].

NF-Y was first identified in *Brassica napus* [6], and has since been identified in other plants, including *Arabidopsis thaliana* [7], *Musa nana* L [8], *Hordeum vulgare* L [9] and *Populus* [10]. It has been revealed that NF-Y executes different biological functions by multitudinous studies recently, including flowering [11], fruit ripening [12] and response to adversity [13], in the processes of plant growth and development. The NF-Y complex regulates the target gene expression either by directly binding the *CCAAT*-containing promoters or by physically interacting and mediating the binding of other proteins that may be transcriptional activators or repressors [14]. A combination of research studies on the structural and functional characteristics manifests the powerful and mysterious regulating effect of the NF-Y gene family in many aspects of plant life.

**Figure 1:**
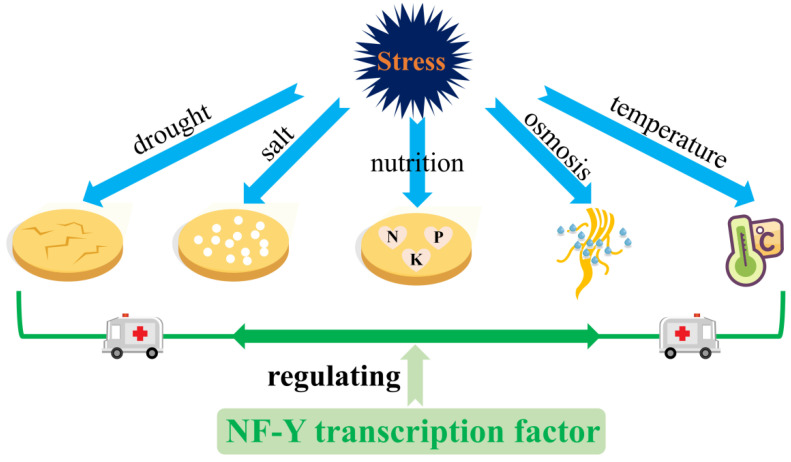
NF-Y transcription factors respond to abiotic stress.

NF-Y is critical to the response to adversity stresses, including abiotic stress, high salt concentrations, drought, high temperature, cold, nutrient deficiency, hypoxia, ultraviolet radiation and heavy metal toxicity. However, these stresses have been reported to negatively affect plant growth, development or crop yield [15,16,17]. To this end, plants have evolved various effective stress-resistance capacities to counteract these stresses. It is noteworthy that some researchers have found that the expression of plant NF-Y is significantly affected in response to abiotic stress [18,19], whereas NF-Y should be one of the pivotal regulators in this process. In recent years, progress has been made on the relationship between plant NF-Y TFs and plant abiotic stress. It is urgent to comprehensively summarize and sort these new results for future studies on NF-Y. Therefore, this work reviews the research progress of the plant NF-Y gene family on its structural characteristics, mechanism of function and its involvement in the plant’s response to abiotic stress, proposing the directions of its function in regulating the abiotic stress response in plants (Figure 1).

## 2. Functional Mechanism of NF-Y Transcription Factor

**Figure 2:**
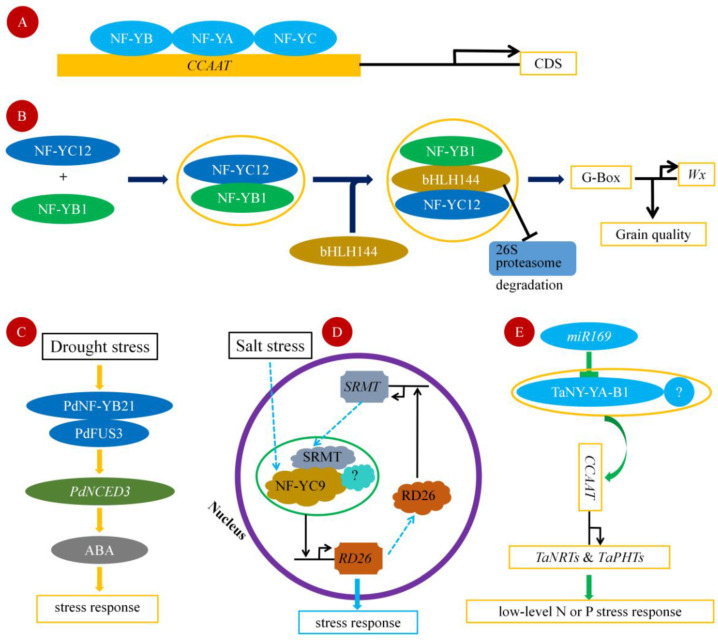
(**A**). A classic NF-Y trimeric complex binds to the *CCAAT* element. (**B**). NF-YB1 binds with NF-YC12 and bHLH144 in order to form a heterotrimer; the trimer complex protects NF-YB1 from the ubiquitin/26S proteasome-mediated degradation. NF-YB1 activates the expression of *Wx* to regulate the starch synthesis by directly binding to the G-box. (**C**). Poplar *PdNF-YB21* responds to drought stress. (**D**). Poplar *NF-YC9* responds to salt stress. (**E**). Wheat *TaNF-YA-B1* responds to low phosphorus and low nitrogen.

The Nuclear Factor-Y (NF-Y), also known as *CCAAT box* binding factor (*CBF*) or Heme Activator Protein (HAP), is a highly conserved trimeric transcription complex that is present in all eukaryotes [20]. Earlier research found that the NF-Y complex is composed of three subunits: NF-YA (also termed *CBF-B* and *HAP2*), NF-YB (*CBF-A* and *HAP3*) and NF-YC (*CBF-C* and *HAP5*). This complex can bind to the *CCAAT* element on the promoter of the target gene to regulate gene expression [21]. The classical NF-Y complex can bind to a promoter containing *CCAAT* sequences to form specific DNA–NF-Y complexes. Any mutation in the *CCAAT* sequence affects the formation of the DNA–NF-Y complex, suggesting that the binding of NF-Y to DNA requires the specific *CCAAT* sequence [1]. However, not all *CCAAT* elements can be recognized and bound by the NF-Y complex, suggesting that the nucleotide sequence and chromatin structure on both sides of *CCAAT* elements may affect the conformation of the NF-Y complex, and thus affect the binding ability of NF-Y complex [22]. Although NF-YA, NF-YB and NF-YC are all required for binding to the *CCAAT box*, it is NF-YA that binds directly to the *CCAAT box*. NF-YB and NF-YC subunit possess histone-fold motifs that allow them to form a heterodimer. After the heterodimer is formed, NF-YB/NF-YC is transported from the cytoplasm to the nucleus where NF-YA is recruited. NF-YA must combine with the NF-YB/NF-YC heterodimer to form a triplex in order to bind to the *CCAAT box*, and then their complex further regulates the transcription of downstream genes (Figure 2A). NF-YB/NF-YC heterodimer binds to the DNA glycophosphate skeleton, which may play a role in stabilizing the protein complex and recognizing specific DNA binding sites [23,24].

However, not all NF-Y regulates downstream gene expression in the form of the NF-YA/NF-YB/NF-YC triplex. In *Arabidopsis*, flowering time regulator CONSTANS (CO/B-BOX PROTEIN 1 BBX1) can bind to NF-YB2/NF-YC3 to form a new triplex structure and bind to flowering regulatory elements. CONSTANS contains a *CCT* domain similar to that of NF-YA but still requires the participation of the NF-YB/NF-YC subunit containing histone folding properties; a triplex comprised of NF-YB2, NF-YC3 and CO binds to the promoter of flowering-promoting gene *FLOWERING LOCUS T*, and the core binding site is the *CCACA* elements [25,26,27,28]. In rice, NF-YB1 binds with NF-YC12 and bHLH144 in order to form a heterotrimer, the trimer complex protects NF-YB1 from the ubiquitin/26S proteasome-mediated degradation. NF-YB1 activates the expression *Wx*, a key granule-bound starch synthase gene, by directly binding to the G-box elements in its promoter and, therefore, regulating starch synthesis [29] (Figure 2B).

## 3. Drought Stress

The abscisic acid (ABA) signaling pathway is essential to plants in the response to drought stress [30,31]. In the past decade, one of the most important advances in plant drought response has been the identification of the ABA receptor and the elucidation of ABA signaling pathways [32,33,34,35]. Recent works have shown that NF-Ys play key roles in regulating ABA signaling pathways; NF-Ys can be involved in regulating ABA synthesis or response to ABA signals, which is known as the ABA-dependent response to drought stress [36,37,38]. A root specific transcription factor *PdNF-YB21* was isolated from *Populus*, which could directly interact with transcription factor *PdFUS3*, and PdFUS3 could directly activate *PdNCED3*, a key gene for ABA synthesis, resulting in a significant increase in root ABA content. Furthermore, ABA enhanced auxin transport in roots, which finally increased root growth and drought resistance. The results showed that *NF-YB21* enhanced the growth and development of poplar under drought by promoting ABA synthesis in roots [16] (Figure 2C). ABA-induced drought responsive transcription factor *PdNF-YB7* was isolated from a fast-growing poplar clone NE-19. The overexpression lines showed a decrease in water loss and an increase in instantaneous leaf water use efficiency (WUE) and leaf water potential; these phenotypes lead to enhanced drought resistance [39]. In well-watered, ABA (100 µM) and dehydration treated vermiculite-grown nine-day-old chickpea seedlings for 2 and 5 h, RNA-seq and RT-qPCR revealed 12 of 18 *CaNF-Y* genes in chickpea in response to ABA in the leaf and root, suggesting that the function of these genes in drought stress may be ABA-dependent [40]. NF-Y regulated plant drought tolerance by regulating the expression of ABA receptor gene *PYR1*. GmNF-YC14, which formed a heterotrimer with GmNF-YA16 and GmNF-YB2, activated the GmPYR1-mediated ABA signal transduction pathway, thereby regulating the drought response in soybean, which was verified by gene knockout and gene overexpression techniques [41]. NF-Y interacted with ABF, a key transcription factor in the ABA pathway. In *Arabidopsis thaliana*, ABF3 and ABF4 could interact with NF-YC3/YC4/YC9. *SOC1* expression in *nf-yc3/yc4/yc9* mutants was significantly reduced by ABA. The response of *nf-yc3/yc4/yc9* to drought was not obvious. Under drought stress, *SOC1* transcription was induced in order to promote flowering under the co-regulation of ABF3/ABF4/NF-Ys, which responded to adversity by shortening the plant’s life span under drought stress [19].

NF-Y enhances plant tolerance to drought by not only regulating the ABA-dependent pathway but also non-ABA dependent activity, mainly through improving plant photosynthetic efficiency, increasing the antioxidant enzymes activity and reducing the content of active hydrogen peroxide [42,43,44,45,46]. Overexpressing *StNF-YC9* in potatoes increased the root length and photosynthetic rate and decreased the water loss rate under short-term drought stress. Under long-term drought stress, the malondialdehyde content decreased, while proline accumulation and the activity of antioxidant enzyme, including superoxide dismutase, catalase and peroxidase, increased. *StNF-YC9* reduced the accumulation of malondialdehyde in potato and played an important role in drought resistance [47]. Two-year-old sweet oranges (Citrus sinensis) were used in the drought stress experiment. The experiment was carried out under greenhouse conditions with the plants grown in plastic pots of 45 L, and *CsNF-YA5* was discovered to play different roles in the leaf and root under water stress. Overexpressing *CsNF-YA5* in tobacco significantly reduced the production of H_2_O_2_ under water stress and increased the photosynthetic rate under normal conditions and drought stress. The biochemical and physiological responses of overexpression lines under drought stress maintained the growth advantage in an environment where soil was water deficient [48]. NF-Y family members *TaNF-YB2* and *TaNF-YC7* were identified in wheat, which could combine with *TaNF-YA7-5B* to form heterotrimers. The expression of *TaNF-YA7-5B* was induced by drought, and the ox*TaNF-YA7-5B* plant could grow and develop normally under dehydration conditions. This was mainly because *TaNF-YA7-5B* regulated stomatal closure, promoted leaf water retention and maintained cell ROS (reactive oxygen species) homeostasis under drought conditions. The expression levels of key regulatory genes *TaCAT1* and *TaPOD4* were positively correlated with the expression levels of *TaNF-YA7-5B* under drought stress, and it was confirmed in previous studies that *TaCAT1* and *TaPOD4* were involved in proline accumulation and ROS clearance. *TaNF-YA7-5B* was an important regulator of drought adaptation in plants, which was independent of ABA [49].

The function of NF-Y in coping with drought stress is expected to be applied in production. In previous studies, scientists confirmed that NF-Y increased the yield of crops, such as wheat, soybeans, corn and rice, in areas suffering from drought or chronic water scarcity [11,29,41,50] and increased biomass accumulation in forestry, such as poplar, apple, spruce and other tree species [16,51,52]. Although NF-Y has been studied for more than 30 years, its function related to drought tolerance has only been discovered in the last 15 years, which may have evolved through the diversification of the gene family encoding the NF-YB subunit. Due to the complexity of plant traits and many influencing factors under drought conditions, NF-Y overexpressed plants show enhanced drought tolerance based on a number of stress-related parameters, including chlorophyll content, stomatal conductance, leaf temperature, reduced wilt and photosynthetic maintenance. Adaptation to these stresses contributes to plant growth advantages in water-deficient environments [53].

## 4. Salt Stress

Salt stress is one of the major adversities for plants. Plants undergo osmotic stress growing in saline–alkali soil [17,54]. Salt stress leads to stunted growth and developmental defects in the plant, including shortened height, obstructed seed germination, hindered reproduction [55,56] and even the demise of the plant [57,58,59]. The function of the NF-Y transcription factor involved in the salt stress response has been discovered through RNA-seq analysis in plants treated with salt [60,61]. The adventitious roots of Jilin ginseng were treated with different concentrations of salt in B5 medium (0, 70, 80, 90 and 100 mM NaCl), and the treated adventitious roots were incubated under dark conditions at 22 °C for 30 days. The expression pattern of NF-Y in *Panax ginseng* suggested that the *PgNF-Y* expression level was different not only among lines, but in temporality and space. The weighted gene co-expression network analysis (WGCNA) indicated that the *PgNF-Y*s function co-ordinately in ginseng. *PgNF-YB9*, *PgNF-YC2* and *PgNF-YC7* responded to salt stress in a synergistic manner [60]. The alfalfa grew for 10 days at 22 °C after treatment with 250 mM NaCl for 0 h, 0.5 h, 1 h, 3 h, 6 h, 12 h and 24 h; the root tips were sampled. The results of RNA-seq suggested that *MsNF-YB2* responded to salt stress at early stages, while *MsNF-YC5* responded to medium term salt stress. Other NF-Ys including *MsNF-YB5*, *MsNF-YB7*, *MsNF-YB15* and *MsNF-YC6* responded to salt stress as well. The WGCNA suggested that *MsNF-YB2* co-expressed with DEAD at the early stages of salt stress, and there are ten genes co-expressed with *MsNF-YC6* coding serine/threonine protein kinases interacting with *CBL*, indicating *MsNF-YC6* might involve in calcium signalling pathway under salt stress [61]. The petunias growing under 25 °C with daily 14-hour light and 10-h darkness were treated with 500 mM NaCl; the samples were taken after 1 h, 3 h, 6 h and 12 h. The RNA-seq revealed that the *PhNF-YA5/6/10* were quickly induced and were highly expressed in the roots. These genes, therefore, might regulate salt tolerance by promoting root development. The expression level of *PhNF-YB3* is relatively higher in the leaf and flower under salt stress. *PhNF-YB3* might play a part in regulating the flowering time and the salt stress response. 12 NF-Y genes, four for each NF-YA, NF-YB and NF-YC, were induced in the root and stem in 30-day-old barley treated with a Hogland solution containing 300 mM NaCl. *NF-YC2* and *NF-YC3* were strongly induced by salt stress and took part in the early plant reaction to stress exposure [9,62].

In recent years, the mechanism of NF-Y*s* in response to salt stress has been reported. *NF-YC9* is located in the cytoplasm under stress-free conditions in poplar, while translocation to the nucleus occurred in response to salt or ABA by interacting with *SRMT*, a *MYB* transcriptional factor. NF-YC9 promoted the expression of SRMT regulated genes, enhancing the salt tolerance in poplar. It is worth noting that the expression level of *NF-YC9* was not induced by salt stress in this process, while the NF-YC9 protein was translocated to the nucleus from the cytoplasm. This phenomenon provides a new way of thinking for the study of the function of NF-Ys [63] (Figure 2D). In maize, it was demonstrated by the technical means of EMSA and yeast that *ZmNF-YA1* promotes the expression of multiple genes that play pivotal roles in response to salt stress and plant development, including *ZmbHLH116*, *ZmPOD64*, *ZmLOX5* and *ZmMBF1c*, under salt stress. *GmNF-YA* is a nuclear factor discovered in soybean, which plays an important role in salt tolerance [64]. *GmNF-YA* interacts with *GmFVE* and weakens the histone deacetylation by reducing the relevance between *GmFVE* and *GmHDA13*. The maintenance of the acetylation level of *GmH3K9* leads to the expression of salt-induced genes [65]. Moreover, NF-Y independently regulates the response to salt stress in wheat. *TaNF-YA10* was identified in salt-tolerant wheat *SR3*, and the heterogeneous expression of *TaNF-YA10* in *Arabidopsis* increased the sensitivity to salt. Further investigation unveiled the independent role *TaNF-YA10* in response to salt stress [66]. Not all NF-Ys that responded to salt positively regulated the response to salt stress. The overexpression of *AtNF-YA1* in *Arabidopsis* prevented the growth of sapling after seed germination under salt stress. Additionally, *ABI3* and *ABI5* is significantly up-regulated along with their target genes *AtEM1* and *AtEM6*, which might restrict the growth of plant [43].

Drought stress along with osmotic stress commonly come with salt stress, thus the response to these two adversities shall be considered when searching for genes involved in salt tolerance. For instance, the ABA signalling pathway plays a key role in response to salt stress since under salt stress more tolerant rootstocks accumulate more ABA [67,68]. The functions of NF-Y family proteins in regulating the response to salt stress are diverse, complicated and cannot be fully understood at this current stage. Most of the study of the NF-Ys function stays in the omics level, and the mechanism of the NF-Y complex is yet to be studied, while most research focuses on the function of a single NF-Y protein. The function of the NF-Y complex is believed to be the priority in the research of the NF-Y family.

## 5. Nutrient Stress

Either insufficient nutrition or overnutrition will cause stress in plants [69,70]. Improving nutrient use efficiency is an important competitive strategy for plants to adapt to barren environments [71]. Studies have shown that NF-Y transcription factors are involved in regulating the absorption and distribution of plant nutrients, including nitrogen absorption, phosphorus absorption, carbon–nutrient balance and coping with low nitrogen and low phosphorus (LNLP) environments [72,73]. QQS (Qua-Quine Starch; *At3g30720*) regulates metabolic processes affecting carbon and nitrogen partitioning to proteins and carbohydrates. Studies have shown that the QQS protein interacts with the transcriptional regulator *AtNF-YC4*. Overexpression of *AtNF-YC4* in *Arabidopsis* mimics the *QQS*-overexpression phenotype, increasing protein and decreasing starch levels; therefore, NF-Y can maintain the homeostasis of plant development by regulating the allocation of carbon and nitrogen in plants [74]. Qu B et al. performed a genome-wide sequence analysis of the A (NF-YA), B (NF-YB) and C (NF-YC) subunits of Nuclear Factor Y (NF-Y) in wheat (*Triticum aestivum*). It was found that most expressions of NF-YAs were positively responsive to low nitrogen and low phosphorus availability, and overexpressing *TaNFYA-B1* significantly increased both nitrogen and phosphorus uptake and grain yield under differing nitrogen and phosphorus supply levels. The increased nitrogen and phosphorus uptake may have resulted from the fact that that overexpressing *TaNFYA-B1* stimulated root development and up-regulated the expression of both nitrate and phosphate transporters in roots. Meanwhile, *TaNFYA-B1* was negatively regulated by *miR169*. Thus, the adaptability of wheat to low nitrogen and phosphorus is enhanced [50] (Figure 2E). A total of 108 NF-Y family members were identified in *B. napus* and categorized into three subfamilies (38 NF-YA, 46 NF-YB and 24 NF-YC). It was found that BnaNF-Ys had different expression patterns under multiple nutrient starvations. Moreover, more *BnaNF-YA* genes were differentially expressed under nutrient limited environments compared to the *BnaNF-YB* and *BnaNF-YC* subfamilies. Among the five rapeseed tissues, 16 *BnaNF-Ys* genes responded diversely to N deprivation [72]. Transcriptome data and RT-qPCR analysis of poplar showed that the expression of NF-YA responded to different forms of nitrogen treatment, and the expression change in the root system was distinctive. NF-YA regulates the response of poplar roots to different forms of nitrogen, indicating that these genes regulate root growth and development [75]. The *AtNF-YA* family can be induced by nutrient stress, similarly nitrogen and phosphorus deficiencies strongly induce the expression of the five *Arabidopsis* NF-YA subfamily members; however, they showed a long-term expression window. It is important that, in its role as a negative regulator, it does so mainly by isolating the recognition of NF-YB/NF-YC heterodimers to other transcription factors that induce the expression of stress response genes [76]. In an analysis of the transcriptomic response to low concentrations of nitrate, the steady-state mRNA levels of *NF-YA5* and other members of the NF-YA family were significantly increased [77]. After 5 weeks of *Arabidopsis* growth, *Arabidopsis* plants were supplied with N-free nutrient solutions to simulate N-deficiency conditions. Nutrient solutions were renewed daily to ensure pH stability. The expression levels of *AtNF-YA2/3/5/8* were significantly up-regulated after 48 h of a nitrogen deficiency culture [78].

The balance of nutrients is essential in plants, especially in short life cycle plants. In field planting, the use of a large number of fertilizers for a long time has led to serious soil pollution, and in forestry production, the long-term nutrient deficiency of plants will lead to tree stuntedness. Therefore, it is particularly important to improve nutrient use efficiency in plants, especially nitrogen and phosphorus [79]. NF-Y TFs play a key role in plant response to nutrient stress, and its function is being continuously explored based on current research, which is expected to make breakthroughs in agroforestry breeding.

## 6. Osmotic Stress

Drought, cold and high salinity cause osmotic stress, which directly affects the development, growth and productivity of plants resulting in crop yield losses [80,81]. The NF-Y transcription factor has been reported to be involved in the regulation of osmotic stress. In peanuts, to analyze the expression pattern of *AhNF-Y* genes, two-week-old seedlings were treated with a nutrient solution containing 200 mM NaCl. The leaves and roots of seedlings treated with NaCl were harvested at 0 h, 4 h, 8 h, 12 h and 16 h. The results revealed that the transcript levels of *AhNF-YA4* and *AhNF-YA8* were down-regulated, and both reached the lowest levels under osmotic stress at approximately 8 h. In contrast, *AhNF-YA11*, *AhNF-YC2* and *AhNF-YC8* had similar expression profiles and showed a trend towards up-regulation. Under osmotic stress, the expression pattern of *AhNF-YA4* and *AhNF-YA11* were different. This difference may be due to the cis-elements in the promoter region [82]. Cultivated with 250 mM mannitol in *Arabidopsis*, overexpression of *NF-YC9* results in osmotic stress hypersensitivity, but the down-regulation of *NF-YC9* expression shows no effect on the osmotic response during post-germination growth. The overexpression of *NF-YC9* enhances the sensitivity to ABA, salt and osmotic stresses during early seedling growth, though the knockdown mutants of *NF-YC9* show wild-type ABA-related phenotypes, suggesting that *NF-YC9* may positively regulate ABA signaling but likely with a functional redundancy. *NF-YC9* interacts with and improves the activities of an ABA responsive bZIP transcription factor *ABI5* and enhances expression of the *ABI5* gene in response to ABA [37]. In addition, apple *MsNF-YB21* was expressed in *Arabidopsis Thaliana*, and it was found that drought stress induced the expression of *MsNF-YB21*, which resulted in root elongation and further increased osmotic-stress tolerance in *Arabidopsis thaliana*. Physiological analysis of the ox*MsNF-YB21* also showed an enhanced antioxidant system. These results provide useful information for further study of the relationship between NF-Ys and osmotic stress in apple [51]. Moreover, after treatment with ABA and exposure to osmotic stress, salt and H_2_O_2_, 27 *PmNF-Y* gene expression profiles were obtained by RT-PCR in *Prunus mume*. It was found that *PmNF-YA1/2/4/5/6*, *PmNF-YB3/4/8/10/11/13* and *PmNF-YC1/2/4/5/6/8* were responsive to ABA and osmotic stress [83]. There are few studies on NF-Ys function regarding osmotic stress, which may be due to the lack of markers on osmotic stress detection. Little is known about the signal perception and signal transduction pathways of osmotic stress in plant cells. The presence of osmotic stress is always accompanied by the presence of other types of stresses, and there are limited studies on the mechanism of osmotic stress.

## 7. Other Stress

Higher plants maintain their growth and development by responding to abiotic stress in various physiological processes. NF-Y TFs are widely involved in the plant’s stress response as monomers, complexes or in combination with other transcription factors. In *Arabidopsis thaliana*, the involvement of the NF-Ys complex in the regulation of ER stress has been clearly elucidated and relies on the complex formed by membrane-associated basic domain/leucine zipper (*bZIP*) transcription factor and NF-Ys: bZIP28 binds to CAGG in the ER stress-response element I (*ERSE-I*) and then binds to *NF-YB3*, *NY-YC2* and *NF-YA4* to form complexes in order to promote the expression of ER stress-induced genes [84]. In studies of the NF-YC family in *Arabidopsis thaliana* and tobacco, *AtNF-YC2* shows an up-regulated expression under oxidative stress conditions. In the study of the necrotizing cell death phenotype that integrates the mechanisms of photooxidative stress accumulation during light exposure, the results showed that reactive oxygen species generated during these photodynamic processes might induce the *NF-YC2* expression [85]. *AtNF-YA2* directly regulates the expression of *AtHSFA3* and *AtHSFA7b* by binding to the promoters, and *AtHSFA7b* regulates the transcription of miRNA169. Moreover, *SlyNF-YA9/A10* are the best orthologs of *AtNF-YA2 in* tomato as it mediates downstream reactions by the same mechanism as in *Arabidopsis*, suggesting that the *SlyNF-YA9/10* and *SlyHSFA7* have conserved functions in heat tolerance in both plant species [86]. In addition, the *Arabidopsis* NF-Y family is involved in the pathway of heat response stress regulation. In this research, a trimer consisting of *NF-YA2*, *NF-YB3* and *DPB3-1* activated the expression of heat stress-induced genes associated with *DREB2A* in protoplasts, and the identified trimer enhanced heat stress-induced gene expression under heat [87]. In the study of the NF-Y family genes in *Sorghum bicolor* L, *NF-YA7* appeared to be associated with high temperature (40 °C) stress, while *NF-YA8* was triggered by both cold (4 °C) and high-temperature stresses [88]. A highly precise and complex role of NF-YA in promoting temperature-induced flowering has been demonstrated, where high environmental-temperature-mediated down-regulation of *miR169hn* leads to the activation of *AtNF-YA2*, which increases the expression of the flowering genes *FT* and *YUCCA2* (*YUC2*) [89]. In cold conditions, *CBF/NF-Y (YZ9)*-overexpressed fruits promoted the coloring in strawberries, suggesting that NF-Y family genes also play a role in cold stress in fleshy fruit [90]. The NF-Y family is also widely involved in vegetative stress. The NF-Y family in *Brassica napus*, which is hyper-sensitive to nitrogen (N) deprivation, was comprehensively identified and systematically characterized 38 NF-YAs, 46 NF-YBs and 24 NF-YCs [72]. 

## 8. Conclusions and Perspectives

Although the NF-Y TFs have been actively studied for decades, the last decade has seen a giant leap in our understanding of their function. The NF-Y family transcription factors have multiple roles in regulating plant growth, development and response to various environmental stresses (Table 1). The functions of the NF-Y family in response to abiotic stress have been extensively studied, mainly focusing on the ABA signaling pathway, particularly related to ABA-mediated drought and salt tolerance. In poplar, the mechanisms of some NF-Ys, including *NF-YB21* and *NF-YC9*, in regulating drought and salt tolerance have been elucidated [16,63]. This is helpful for the application of NF-Ys in production practice, especially in molecular breeding, and has great reference significance.

Due to the complexity of the NF-Y complex, the signaling pathways and regulatory models led by the NF-Y complex are still poorly understood. It is still necessary to further investigate the mechanisms of NY-Ys responses to abiotic stress in plant hormones (auxin, cytokinin, ethylene and brassinolide) dependent pathways and the role in other stress responses. It is generally believed that the complex formed with NF-YA is directly bound to the *CCAAT box*. However, recent studies have demonstarted that NF-Ys interact with other transcription factors. Since NF-YBs/YCs generally cannot bind directly to promoters and the target is determined by the transcription factors they interact with, more potential targets for NF-Ys should exist. Plants will have complex physiological changes in response to stress, and there are many genes involved in both sensing stress signals and responding to stress signals. However, the way in which NF-Ys sense and respond to stress signals is currently unclear. Most studies of NF-Ys have not reported how NF-Ys bind to promoters. Moreover, the majority of them are in response to drought, salt, temperature stress and nutrient stress. There are some general environmental factors, without specific regulatory mechanisms, which also lead to our lack of understanding of NF-Ys. The reason for this may be that NF-Ys have strong interactions among themselves, and NF-Ys interacting with other genes or TFs produce additional complexes, making these questions extremely complicated. There is an urgent need to decipher the role of NF-Ys in diverse physiological processes in plants and a theoretical basis is needed to address critical problems regarding plant production and resistance. Many studies have reported the existence of different abiotic stress responsive cis-elements in NF-Y promoters, and understanding each TF’s specific regulation of these cis-elements is critical for stress mitigation in plant improvement projects, which helps to achieve the agriculture and forestry sustainability and food security for the growing world population.

## Figures and Tables

**Table 1:** NF-Y transcription factors (TFs) involved in abiotic stress responses in plants.

Gene	Species	Phenotypic Manifestation	References
*PdNF-YB21*	*Populus*	Enhanced drought tolerance and promoted root development.	[16]
*PdNF-YB7*	*Populus*	Enhanced sensitivity to ABA and reduced water loss.	[39]
*GmNF-YA16/YB2/YC14*	*Glycine max* L.	Enhanced tolerance to drought stress and increased sensitivity to ABA.	[41]
*AtNF-YC3/YC4/YC9*	*Arabidopsis thaliana*	Enhanced drought tolerance and promoted flowering.	[19]
*StNF-YC9*	*Solanum tuberosum* L.	Better growth under drought.	[47]
*CsNF-YA5*	*Citrus sinensis*	Enhanced leaf water retention capacity.	[48]
*TaNF-YA7/YB2/YC7*	*Triticum aestivum* L.	Regulated stomatal closure, enhanced leaf water retention capacity and maintained cell ROS homeostasis.	[49]
*PgNF-YB9/C2/C7*	*Panax ginseng*	Response to salt stress.	[60]
*MsNF-YB2*	*Medicago* L.	Regulation of Ca^2+^ signaling pathway under salt stress.	[61]
*NF-YC9*	*Populus*	Interaction with MYB transcription factor enhances salt tolerance of poplar.	[63]
*ZmNF-YA1*	*Zea mays* L.	Activate the salt tolerance gene.	[64]
*TaNF-YA10*	*Triticum aestivum* L.	Enhanced plant sensitivity to salinity.	[66]
*AtNF-YA1*	*Arabidopsis thaliana*	Negative regulation of plant development under salt stress.	[43]
*GmNF-YA1/YB1*	*Glycine max* L.	Provide carbohydrates.	[50]
*GmNF-YC4*	*Glycine max* L.	Increased protein level and reduced starch content.	[74]
*AtNF-YA4/YB3/YC2*	*Arabidopsis thaliana*	Up-regulated the expression of ER stress-induced genes.	[84]
*AtNF-YA2/YB3*	*Arabidopsis thaliana*	Regulation of plant heat tolerance.	[87]
*Sb* *NF-YA7/A8*	*Sorghum bicolor* L.	Response temperature change.	[88]
